# Mitochondrial Ca^2+^ Remodeling is a Prime Factor in Oncogenic Behavior

**DOI:** 10.3389/fonc.2015.00143

**Published:** 2015-06-25

**Authors:** Alessandro Rimessi, Simone Patergnani, Massimo Bonora, Mariusz R. Wieckowski, Paolo Pinton

**Affiliations:** ^1^Section of Pathology, Oncology and Experimental Biology, Laboratory for Technologies of Advanced Therapies (LTTA), Department of Morphology, Surgery and Experimental Medicine, University of Ferrara, Ferrara, Italy; ^2^Department of Biochemistry, Nencki Institute of Experimental Biology, Warsaw, Poland

**Keywords:** mitochondrial dysfunction, cancer, Ca^2+^ signaling, oncogene and oncosuppressor

## Abstract

Cancer is sustained by defects in the mechanisms underlying cell proliferation, mitochondrial metabolism, and cell death. Mitochondrial Ca^2+^ ions are central to all these processes, serving as signaling molecules with specific spatial localization, magnitude, and temporal characteristics. Mutations in mtDNA, aberrant expression and/or regulation of Ca^2+^-handling/transport proteins and abnormal Ca^2+^-dependent relationships among the cytosol, endoplasmic reticulum, and mitochondria can cause the deregulation of mitochondrial Ca^2+^-dependent pathways that are related to these processes, thus determining oncogenic behavior. In this review, we propose that mitochondrial Ca^2+^ remodeling plays a pivotal role in shaping the oncogenic signaling cascade, which is a required step for cancer formation and maintenance. We will describe recent studies that highlight the importance of mitochondria in inducing pivotal “cancer hallmarks” and discuss possible tools to manipulate mitochondrial Ca^2+^ to modulate cancer survival.

## Introduction

The mitochondrion is an endosymbiotic organelle that characterizes any eukaryotic cell, participating in many aspects of cell physiology, such as ATP production (1), intracellular Ca^2+^ signaling (2), lipid metabolism (3), reactive oxygen species (ROS) production (4), inflammasome activation (5), and cell death regulation (6).

Mitochondria can fulfill a large energy demand due to their biosynthetic capacities; during mitochondrial respiration, it can rapidly accumulate Ca^2+^ through an electrogenic pathway (7) and via their close apposition with the Ca^2+^-storing endoplasmic reticulum (ER) (8). Ca^2+^ released from the ER generates microdomains consisting of high [Ca^2+^] that are sequestered by the mitochondrial Ca^2+^ uniporter (MCU) (9, 10).

**Figure 1:**
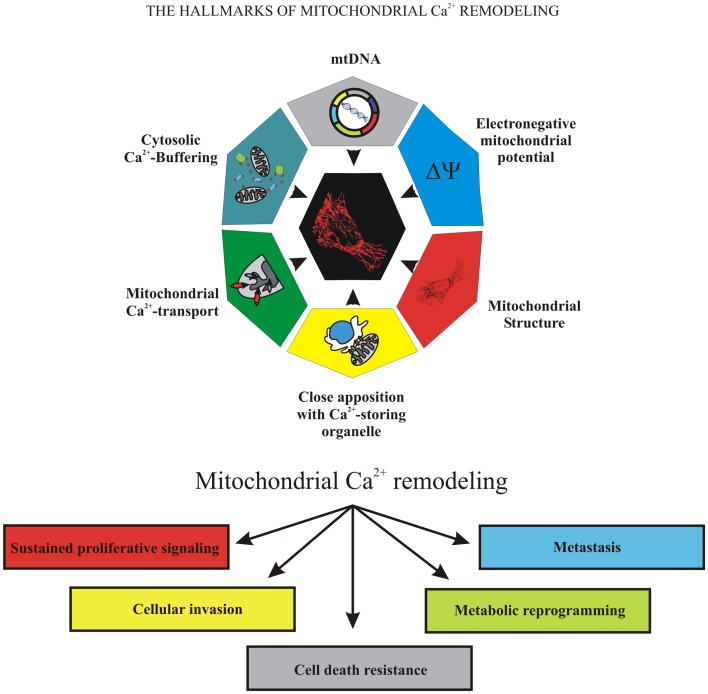
**The hallmarks of mitochondrial Ca^2+^ remodeling**. This illustration encompasses the six hallmarks of remodeled mitochondrial Ca^2+^ signaling that are involved in pathogenesis of some and perhaps all cancers. Uncontrolled growth, programed cell death evasion, metabolic reprograming, invasion, and metastasis are the “cancer hallmarks” that are linked to mitochondrial Ca^2+^ remodeling, the prime reason of oncogenic behavior.

Because of the important role of mitochondria in cell fate, many pathological conditions are associated with mitochondrial failure, including metabolic and neurodegenerative diseases (11), inflammation, and cancer (12). Mitochondrial dysfunction is associated with cancer development and progression. During carcinogenesis in some malignant cells, the mitochondrial Ca^2+^ signaling was significantly remodeled, compromising mitochondrial functions in ways that caused them to overwhelm normal cells. Uncontrolled growth, programed cell death evasion, metabolic reprograming, invasion, and metastasis are “cancer hallmarks” that are linked to mitochondrial Ca^2+^ remodeling, which could be the prime reason for these transformations (Figure 1).

In this review, we will describe the current knowledge regarding the role of mitochondrial Ca^2+^ signaling in cancer and the rationale for targeting cancer cells with mitochondrial Ca^2+^ modulators as a pharmacological therapeutic strategy.

## Mitochondrial Ca^2+^ Homeostasis

Due to their strong electronegative potential (ΔΨ) and by topological organization, mitochondria are able to accumulate Ca^2+^ in the mitochondrial matrix. This organelle can generate contact sites with the ER (13), where the channels responsible for Ca^2+^-release (inositol trisphosphate receptor, IP3R) are juxtaposed to mitochondrial Ca^2+^ handling/transport (14). Voltage-dependent anion selective channels (VDACs) are permeable to Ca^2+^ and are in the outer mitochondrial membrane (OMM), toward the matrix-specific Ca^2+^ transporter, where these channels participate in structures devoted functionally to facilitating Ca^2+^-flow toward the mitochondrion (15). This flow allows the generation of a high [Ca^2+^] microdomain that is sufficient to trigger the opening of mitochondrial uniporter complex (16). MCU is a two-transmembrane domain protein that spans across the mitochondrial inner membrane, oligomerizing it to form the Ca^2+^-channel portion of the complex (9, 10), and where it can accommodate the MCU paralog MCUb. This protein does not exhibit any channel-forming activity and may behave as a negative regulator of MCU complex (17). MCU/MCUb oligomers physically interact with two regulators located in the intermembrane space, MICU1 and MICU2. Silencing studies have indicated that both regulators participate in setting the Ca^2+^-threshold for mitochondrial Ca^2+^-uptake (18–20). Finally, the MCU regulator EMRE mediates the interaction between MCU oligomers and MICU1/MICU2 (16).

The accumulation of Ca^2+^ requires efficient mechanisms to turn off the signal to prevent the mitochondrial permeability transition pore (mPTP) opening. The most well-characterized Ca^2+^-efflux mechanism involves the mitochondrial Na^+^/Ca^2+^ exchanger (21). This protein forms dimers that transport either Na^+^ or Li^+^ in exchange for Ca^2+^. Then, accumulated Na^+^ is extruded via mitochondrial Na^+^/H^+^ exchange. This activity induces the dissipation of ΔΨ, causing mitochondrial Ca^2+^-efflux (22). In contrast, a non-electrogenic Ca^2+^/2H^+^ exchange has been proposed to be responsible for Ca^2+^-efflux in non-excitable cells. Its molecular identity has been proposed to be leucine zipper-EF-hand containing transmembrane protein 1 (LETM1) (23). Ongoing discussions have proposed that mPTP could be a component of the Ca^2+^-efflux system due to the involvement of a non-specific channel that has been identified as the C subunit of mitochondrial F1/FO ATP synthase (24). The manipulation of C subunit expression is able to promote or inhibit mPTP opening without affecting the physiological mitochondrial Ca^2+^ homeostasis (25).

## Mitochondrial Ca^2+^ Homeostasis during Proliferation and Cell Death

Cancer is sustained by defects in the mechanisms underlying cell proliferation and cell death, and mitochondrial Ca^2+^ ions are central to both events (Figure 1).

To meet the increased energy demand of cells during proliferation, mitochondria utilize fundamental pathways for energy metabolism, including the citric acid cycle (TCA) and oxidative phosphorylation. An increase in mitochondrial ATP levels was shown to occur in parallel with an evoked increase in mitochondrial [Ca^2+^] (26). The first results were obtained by Denton and McCormack, who showed that mitochondrial Ca^2+^-activated dehydrogenase enzymes led to increased NADH and thus ATP production (27, 28). The involvement of different mitochondrial targets in the regulation of oxidative phosphorylation by Ca^2+^, including dehydrogenase activity, F1-F0-ATPase, and mitochondrial substrate-transport has been proposed (29).

Three are the mitochondrial dehydrogenase sensitive to Ca^2+^ of TCA: Pyruvate dehydrogenase (PDH), isocitrate dehydrogenase and α-ketoglutarate dehydrogenase. All of these molecules exhibit different Ca^2+^-dependent mechanisms, suggesting that the origins or specific activation signals are quite dissimilar. Although the deregulation of mitochondrial metabolism is intimately linked to oncogenic behavior, the role of cellular metabolism in oncogene-induced transformation is unclear. Recently, the mitochondrial gatekeeper PDH was shown to be a crucial mediator of oncogene-induced senescence promoted by BRAF (V600E), which is the oncogene that is commonly mutated in tumors (30).

The efficient mitochondrial respiration and maintenance of normal cell bioenergetics require basal constitutive low-level Ca^2+^ signaling to sustain the mitochondrial Ca^2+^-uptake provided by IP3R (31). The enhanced resistance to apoptosis of cancer cells may involve its downregulated expression or activation, compromising IP3R-mediated Ca^2+^ release. Indeed, in bladder cancer cells, the acquisition of cisplatin resistance was sustained by the drug-induced downregulation of IP3R1 expression (32). Additionally, the disruption of IP3R-mediated ER–mitochondria crosstalk compromised the production of mitochondrial ATP, increasing the AMP/ATP ratio, which subsequently activated the energy sensor AMP-activated protein kinase, inducing autophagy (33, 34). This cascade of events occurs in advanced cancer under hypoxic and nutrient-deficient conditions that activate autophagy as a pro-survival mechanism (35).

The failure of mitochondria to take-up Ca^2+^ results in a failed intracellular Ca^2+^ buffering with consequent aberrant activation of cytosolic Ca^2+^-dependent enzymes, such as calpain proteases (36) and calmodulin-dependent kinases (37). In turn, these enzymes alter cellular signaling cascades with consequent effects on cell growth (38) or lead to increased glycolysis and tumor cell invasion (39). However, the activation of calcineurin protein phosphatase by increased cytosolic Ca^2+^ leads to the dephosphorylation of IκBβ and successive NF-κB activation (40), which promote apoptosis resistance and cell migration (41). The migratory capacity of tumor cells is linked with mitochondria morphology (42, 43). In breast cancer, higher fragmented mitochondria content correlates with metastatic potential and the cell’s ability to migrate and invade is significantly reduced when the Dynamin-related protein 1 is silenced. The relationship between mitochondrial shaping and Ca^2+^ is well represented by the involvement of Rho-GTPase Miro and by Drp1 [as reviewed in Ref. (44, 45)].

Endoplasmic reticulum–mitochondria contact sites allow the accumulation of Ca^2+^ in response to not only physiological agonists but also in response to apoptotic stimuli that induce ER Ca^2+^-release (46). While moderate Ca^2+^ levels are essential for normal mitochondrial activities, mitochondrial Ca^2+^ overload is detrimental to mitochondrial morphology, causing mitochondrial permeabilization and organelle swelling, with the consequent release of pro-apoptotic factors (6). Recently, mitochondrial Ca^2+^ overload has also been implicated in mitophagy, the selective degradation of damaged mitochondria (45).

A critical link between Ca^2+^ and apoptosis was established by the oncoprotein Bcl-2. Evidence show that it reduces the filling state of intracellular Ca^2+^-stores, reducing the Ca^2+^ signaling evoked by physiological and pathological stimuli, but this observation is still debated (47, 48). Bcl-2 is also a critical regulator of IP3R and interacts directly with this molecule to inhibit channel opening and ER Ca^2+^-release, thereby assuring Ca^2+^-dependent cell proliferation and apoptosis protection (49, 50). The mechanism by which Bcl-2 family members control apoptosis remains unclear; however, their activity involves controlling OMM permeability, which also requires interactions with OMM proteins such as VDACs (51). Pro-apoptotic proteins of the Bcl-2 family (such as Bax) exert opposite effects (i.e., a potentiation of Ca^2+^-mediated signals) counteracting the effects of Bcl-2 on ER Ca^2+^ filling (52).

Voltage-dependent anion-selective channels are readily permeable to Ca^2+^ and can interact with IP3Rs at the ER (53). VDACs are expressed in three isoforms that have common channeling properties but different roles in cell survival (54, 55). Although all three VDAC isoforms are equivalent in facilitating mitochondrial Ca^2+^-uptake upon agonist stimulation, VDAC1 is preferentially involved in the transmission of low-amplitude apoptotic Ca^2+^ signals to mitochondria. VDAC1 gene expression levels are predictors of poor outcome in NSCLC and in other cancers (56). VDACs are subjected to multiple levels of regulation, including expression levels, post-translational modification, and protein–protein interactions, all of which can limit Ca^2+^-uptake. For example, VDAC may inhibit apoptosis, promoting tumorigenesis and glycolysis through specific interactions with hexokinase-2 (HK2). The upregulation of HK expression in tumor cells and its binding to VDAC provide both metabolic benefits and apoptosis-suppressive capacity, offering advantages in terms of growth and resistance to chemotherapy (57, 58). Mitochondrial HK2 interferes with the ability of Bax to bind to mitochondria and release cytochrome *c*, thus antagonizing cell death (59).

Mitochondria are also the critical sites for “apoptotic” Ca^2+^ signaling due to MCU, which is responsible for low-affinity Ca^2+^-uptake, influencing a myriad of Ca^2+^-dependent processes, including cell death (60). MCU-overexpressing cells treated with apoptotic stimuli, such as H_2_O_2_ and ceramide, exhibited more pronounced apoptotic responses (10), while the overexpression of a MCU-targeting microRNA miR-25 in colon cancer cells impaired mitochondrial Ca^2+^-uptake and increased apoptosis resistance (61). Thus, MCU appears to be a crucial protein for tumorigenesis, and its specific pharmacological activators, if identified, could become a useful tool.

Much remains to be understood regarding the additional signals that converge on mitochondria and switch their function to apoptotic inducers. These organelles can handle large Ca^2+^ loads under physiological conditions (e.g., in cardiac myocytes, significant amounts of Ca^2+^ accumulate in mitochondria with every heartbeat) with no deleterious effects. Oxidative stress is considered an important factor for additional “apoptotic signal” (62, 63). ROS is involved in the regulation of physiological processes but may also be harmful if produced excessively by oxidative phosphorylation. A feature of transformed cells is increased mitochondrial ROS levels, which are attributed to inefficiencies in electron transport at the respiratory chain, increased metabolic demand, reduced ROS scavenging, oncogene-induced replicative stress, and altered mitochondrial dynamics (64, 65). Indeed, ROS promote tumorigenesis in numerous ways, including the stabilization of hypoxia-inducible factor HIF-α, the induction of oxidative base damage to mtDNA, the activation of both NRF2 and NF-kB transcription factors, and the alteration of Ca^2+^ flux (66).

## Alterations to Mitochondrial Ca^2+^ Homeostasis in Cancer Cells

A growing number of tumor suppressor genes and oncogenes have been investigated due to their ability to regulate mitochondrial function, influencing the physiological processes of this organelle and its capacity to uptake Ca^2+^.

Among these genes, increasing evidence has highlighted the role of the oncogene Ras in the growth and maintenance of the tumor environment (67). The first link between mitochondria and Ras is represented by the direct translocation of Ras into the organelle, where the phosphorylated form exerts apoptotic activity (68). Although some evidence has suggested a possible role of mitochondria in the regulation of Ras-driven modifications, a clear molecular mechanism underlying this relationship has not yet been addressed. The oncogenic function of Ras facilitates Stat3 translocation to mitochondria, which promotes mtDNA transcription (69), and Ras-dependent mitochondrial dysfunction causes metabolic changes and ROS, which promote tumor development (70). A direct link between Ca^2+^ regulation and oncogenic Ras has been shown by Rimessi et al., in which H-Ras affects the correct transmission of Ca^2+^ waves from the ER to mitochondria (71). As a result, mitochondrial metabolism and apoptosis are deeply compromised, and a neoplastic phenotype is induced. Recently, cancer stem cells in pancreatic cancer were shown to survive oncogenic K-Ras ablation by relying on oxidative phosphorylation (72) and by exhibiting high sensitivity to oxidative phosphorylation inhibitors to prevent tumors. This finding is consistent with other recent reports referring to leukemia cancer cells (73–75).

Additionally, the oncogene AKT regulates cell growth and apoptosis, and upon PI3K stimulation, it rapidly accumulates in mitochondria (76), where it inhibits apoptosis in an HK-dependent manner (77, 78). AKT phosphorylates several pro-apoptotic proteins, such as Bad and Bax, inhibiting their cell death functions. AKT also phosphorylates HK2, promoting binding with VDAC1, and preventing a Ca^2+^-dependent apoptotic response (79). In addition, AKT phosphorylates the IP3R isoforms that inhibit ER Ca^2+^ release and apoptosis, avoiding mitochondrial Ca^2+^ overload (80), preferentially phosphorylating the IP3R3 (81). AKT is intimately regulated by the tumor suppressor protein phosphatase and tensin homolog (PTEN), commonly lost or mutated in human cancers (82). PTEN was demonstrated to perform its growth-attenuating activity by regulating ER Ca^2+^ release via IP3R3. In this study, PTEN was shown to directly interact with IP3R, counteracting the reduced IP3R-dependent Ca^2+^ release mediated by AKT phosphorylation in a phosphatase-dependent manner (83). Another tumor suppressor that found to downregulate the anti-apoptotic features of AKT is PML. This tumor suppressor, where its tumoral behavior is regulated by PIAS1 and SUMOylation machinery (84), promotes the formation of a multiprotein complex containing IP3R3, AKT, and the protein phosphatase PP2a, orchestrating ER–mitochondria Ca^2+^ flux (85). An other example is the tumor suppressor BRCA1, recruited to the ER during apoptosis in an IP3R-dependent manner, sensitizing the IP3R to its ligand (86).

Additionally, the tumor suppressor p53 can regulate tumorigenesis in a Ca^2+^-dependent pathway. Different reports have documented the ability of p53 to regulate cell fate via post-translational modifications (87, 88); however, p53 was recently shown to function in modulating Ca^2+^ transfer from ER to mitochondria. An extranuclear pool of p53 interacts with the Ca^2+^-ATPase SERCA, modulating Ca^2+^-dependent ER–mitochondria crosstalk, mitochondrial swelling and apoptosis induction (89, 90).

The tumor suppressor FHIT functions in a different manner. This hydrolase, which acts as a tumor suppressor *in vivo* and *in vitro*, enhances apoptosis susceptibility by acting directly on the mitochondrial compartment. Specifically, FHIT increases the affinity of the mitochondrial machinery for physiological agonist- and apoptotic challenge-triggered Ca^2+^-uptake into mitochondria (91).

## Anti-Cancer Drugs Act on Mitochondrial Ca^2+^ Homeostasis

The Ca^2+^ ion regulates various cellular processes, some of which are involved in tumorigenesis and which are thus considered as attractive drug targets for cancer therapy. Mitochondrial Ca^2+^ homeostasis may be affected by anti-cancer drugs that directly influence mitochondria or targets that indirectly regulate mitochondrial Ca^2+^-uptake. Some of these drugs may affect mitochondrial Ca^2+^ uptake due to the depolarization of ΔΨ. The compounds included in this category are as follows: pancratistatin (a natural alkaloid exhibiting anti-cancer effects against human colorectal adenocarcinoma xenografts (92) and colorectal carcinoma cell lines, rhodamine-123 [the anti-cancer effects of rhodamine-123 can be explained by its higher retention in kidney and breast cancer cells compared to non-tumorigenic cells (93, 94)], a naphthyridine derivative [4-phenyl-2,7-di(piperazin-1-yl)-1,8-naphthyridine (95)], PMT7 [redox-active quinone phloroglucinol derivative (96)], edelfosine [1-*O*-octadecyl-2-*O*-methyl-racglycero-3-phosphocholine; its redistribution to the mitochondria in HeLa cells causes mitochondrial depolarization and apoptosis (97)], and many others that inhibit the mitochondrial respiratory chain that have been reviewed in detail by Wen et al. (98).

Anti-cancer drugs can also influence the mitochondrial Ca^2+^-uptake indirectly through the induction of ER stress. For example, tunicamycin induces the accumulation of unfolded proteins in the ER, which is known to enhance antitumor effects of different chemotherapy drugs *in vitro* and *in vivo* (99). The cannabinoids induce apoptosis of pancreatic tumor cells via the ER-stress-related genes activating transcription factor 4 and TRB3 (100), or by the photo-oxidative treatment with hypericin that causes ER stress, induction of UPR target genes, and depletion of ER Ca^2+^-stores (101). Brefeldin A blocks protein transport from the ER to the Golgi apparatus (102), causes the deregulation of ER Ca^2+^ homeostasis, or affects IP3R activity. Bortezomib, a selective proteasome inhibitor approved for use in patients with multiple myeloma, requires the mitochondrial uniporter as critical regulatory factor for its cytotoxicity (103).

The anti-apoptotic Ca^2+^ effects of Bcl-2 are attributable to direct interactions with IP3R through the BH4 domain (50, 104). A peptide covering this residue was used to disrupt the IP3R/Bcl-2-protein complex, enhancing the Ca^2+^-dependent apoptotic response in a variety of tumoral cells (105–108).

The HK–VDAC complex is also an oncological target of erastin, which is an antitumor agent that is selective for tumor cells bearing oncogenic Ras (109), and of the chemo-potentiator 3-bromopyruvate, which is an inhibitor of energy metabolism in tumor cells (109, 110). Clotrimazole and bifonazole are also effective (111, 112), as is the anti-cancer agent furano naphthoquinone, which induces a caspase-dependent apoptosis pathway through ROS production (113).

The most direct effects of potential anti-cancer drugs on mitochondrial Ca^2+^-uptake were obtained by potentiating MCU complex expression/activity. Unfortunately, recent molecular discoveries have impeded the identification of an MCU-specific inducer. Alternative approaches could include MCU-targeting anti-miRNA-based therapy and anti-miRNA 25 oligonucleotides that could be used as potential agents against cancer (114).

## Conclusion

“Uncontrolled growth, programed cell death evasion, metabolic reprograming, invasion, and metastasis are ‘cancer hallmarks’ that are linked to mitochondrial Ca^2+^ remodeling, which could be the prime reason for these transformations.” This speculation in the Section “Introduction” is sustained by the knowledge described thus far and by the most recent discoveries that highlight the role of oxidative phosphorylation in tumor progression and maintenance. Previously, mitochondrial dysfunction has been considered a consequence rather than a key player in tumor development. Recently, cancer stem cells were shown to survive oncogenic ablation by relying on oxidative phosphorylation (72), and only mtDNA-depleted cancer cells were capable of recovering mtDNA from host-formed metastasizing cancers *in vivo* (115), thus highlighting the malicious capacity of tumor cells to acquire mtDNA from normal bystander cells. Considering that the driving force for mitochondrial Ca^2+^ internalization is ΔΨ, which is generated by respiratory chain, mitochondrial Ca^2+^ signaling has become a prime factor that is responsible for oncogenic behavior. Although mitochondrial Ca^2+^ remains a novel area of research in oncology, considerable further work is required to clarify the molecular mechanisms by which it contributes to cancer formation and maintenance, and greater knowledge of this target may be of importance in the development of new therapeutic strategies.

## Conflict of Interest Statement

The authors declare that the research was conducted in the absence of any commercial or financial relationships that could be construed as a potential conflict of interest.
